# A Multifunctional Trypsin Protease Inhibitor from Yellow Bell Pepper Seeds: Uncovering Its Dual Antifungal and Hypoglycemic Properties

**DOI:** 10.3390/pharmaceutics15030781

**Published:** 2023-02-27

**Authors:** Juliana Cotabarren, Brenda Ozón, Santiago Claver, Florencia Geier, Martina Rossotti, Javier Garcia-Pardo, Walter David Obregón

**Affiliations:** 1 Centro de Investigación de Proteínas Vegetales (CIPROVE), Departamento de Ciencias Biológicas, Facultad de Ciencias Exactas, Universidad Nacional de la Plata, 47 y 115 s/N, La Plata B1900AVW, Buenos Aires, Argentina; 2 Departament de Bioquimica i Biologia Molecular, Institut de Biotecnologia i Biomedicina, Universitat Autònoma de Barcelona, Bellaterra, 08193 Barcelona, Spain

**Keywords:** antifungal, bioactive compound, bioactive peptide, *Capsicum annuum* L., *Candida albicans*, protease, protease inhibitor, phytocystatin, trypsin inhibitor, natural product

## Abstract

Fungal infections are a growing public health concern worldwide and the emergence of antifungal resistance has limited the number of therapeutic options. Therefore, developing novel strategies for identifying and developing new antifungal compounds is an active area of research in the pharmaceutical industry. In this study, we purified and characterized a trypsin protease inhibitor obtained from Yellow Bell Pepper (*Capsicum annuum* L.) seeds. The inhibitor not only showed potent and specific activity against the pathogenic fungus *Candida albicans*, but was also found to be non-toxic against human cells. Furthermore, this inhibitor is unique in that it also inhibits α-1,4-glucosidase, positioning it as one of the first plant-derived protease inhibitors with dual biological activity. This exciting discovery opens new avenues for the development of this inhibitor as a promising antifungal agent and highlights the potential of plant-derived protease inhibitors as a rich source for the discovery of novel multifunctional bioactive molecules.

## 1. Introduction

In the last decade, the increase in antifungal resistance among pathogenic fungi has become a serious concern worldwide. Recent studies have suggested that pathogenic fungi are responsible of over 150 million severe fungal infections and about 1.7 million infectious-disease related deaths annually [1]. Fungi, which can be unicellular or multicellular eukaryotic organisms, can survive in a wide range of environmental conditions. Due to their eukaryotic nature, the number of currently available drugs to treat invasive fungal infections is limited to a reduced number of chemical entities [2]. These compounds perform their actions via three different main mechanisms: (I) inhibiting ergosterol biosynthesis and/or its availability, (II) inhibiting the DNA/RNA biosynthesis, and (III) inhibiting fungal cell wall biosynthesis and membrane sterols. The irresponsible use of antifungals has led to the emergence of increasingly resistant pathogenic strains, leaving very few therapeutic options to treat aggressive fungal infections. Therefore, developing novel strategies for identifying and developing novel antifungal compounds is an active area of research in the pharmaceutical industry.

One promising strategy to develop novel antifungals is targeting their virulence factors [3]. Many pathogenic fungi produce molecules that facilitate the adhesion to the host tissues. Another common virulence strategy is the production and secretion of proteases, such as aspartic and serine proteases, which allows pathogenic fungi to survive and penetrate host tissues during infection. Given the relevant role of extracellular fungal proteases, it has been speculated that specific protease inhibitors (PIs) may be a promising strategy for the development of novel efficient antifungal drugs.

Plants have traditionally been a crucial source for identifying and developing new molecules with antifungal properties. In recent years, medicinal plants and plant-derived extracts have been used as important sources for antifungal drug discovery [4,5]. Higher plants possess the ability to produce a wide variety of secondary metabolites with high chemical diversity, including proteins, peptides, sugars, and nucleosides. Many of these compounds, such as plant-derived proteins and peptides, are PIs with demonstrated antibacterial [6,7] and antifungal properties [8]. Plants are rich in small proteinaceous PIs as these molecules are involved in plant defense mechanisms against pathogens and predators [5,9,10]. In addition, plant proteins contain a relatively high content of disulfide bridges, which allows plant-derived PIs to effectively slow or inhibit the catalytic action of their target enzymes, even under extreme environmental conditions [5]. Compared to small chemical molecules, natural inhibitors are often less toxic, which leads to better tolerability. In addition, these proteins have showed a remarkable multifunctionality. Such properties can be exploited for antifungal drug development.

In this study, we report the isolation and characterization of a thermostable trypsin protease inhibitor obtained from Yellow Bell Pepper (*Capsicum annuum* L.) seeds. We have evaluated the inhibitory capacity against a set of pathogenic bacteria and fungi. Ultimately, this inhibitor showed both potent and specific activity in vitro against pathogenic *Candida albicans* and hypoglycemic activity. Furthermore, our studies also suggest that this naturally occurring molecule is safe and has no toxicity against human cells. Taken together, the current results demonstrate that this novel inhibitor could effectively be used as an antifungal agent in pharmaceutical preparations to prevent invasive candida infections.

## 2. Materials and Methods

### 2.1. Materials

The seeds of the Yellow Bell Pepper (*Capsicum annuum*) were hand-collected from local farmers around La Plata, Buenos Aires, Argentina. The seeds were processed as previously described elsewhere [11]. The reagents sodium chloride, Coomassie Blue G-250, N,N,N′,N′-tetramethylethylenediamine (TEMED), tris (hydroxymethyl) aminomethane, sodium dodecyl sulphate (SDS), bovine serum albumin (BSA), β-mercaptoethanol (β ME), Nα-benzoyl-DL-arginine-p-nitroanilide (BApNA), and 4-nitrophenol-α-D-glucopyranoside (PNPG) were purchased from Sigma-Aldrich (San Luis, MO, USA). The proteases used in this study, such as Trypsin and α-Glucosidase, were obtained from Sigma-Aldrich. To perform PI purification, a Glyoxyl-agarose resin was obtained from FlukaTM. Fluconazole was purchased from Signa-Aldrich. All chemicals and reagents used in this study were of analytical grade, unless otherwise specified.

### 2.2. Cell Lines and Pathogenic Microbial Strains

*Escherichia coli* (ATCC 25923), *Pseudomonas aeruginosa* (ATCC 27853), *Staphylococcus* aureus (ATCC 29213), *Klebsiella pneumoniae* (ATCC 700603), *Enterococcus faecalis* (ATCC 29212), *Candida albicans* (CIPROVE), *Candida tropicalis* (CIPROVE), *Candida glabrata* (CIPROVE), *Candida krusei* (CIPROVE), *Rhodotorula* spp. (CIPROVE) and *Saccharomyces cerevisiae* (CIPROVE) were obtained from CIPROVE.

### 2.3. Crude Extract Preparation

To prepare the extracts, *Capsicum annuum* seeds were washed with distilled water and stored at −20 °C until PI extraction. *Capsicum annuum* dry seeds (about 30 g) were ground using a mechanic blender. The sample was grounded with 100 mL of 0.01 M phosphate buffer, 0.1 M NaCl, pH 7.4 placed in an ice bath to avoid possible protein denaturation. Afterwards, the mixture was incubated 3 h at 4 °C and filtered using a gauze. The resultant homogenate was centrifuged at 7000× *g* for 30 min at 4 °C, and the supernatant (from now on: YBPCE) was collected and frozen at −20 °C until further processing. The total protein content present in the sample was as described in our previous publication [12] using the Bradford’s assay [13]. Bovine serum albumin (BSA) was used as standard (0.1 mg/mL). Next, the trypsin inhibitory activity present in the clarified homogenate was determined as described below.

### 2.4. Trypsin Inhibition Measurements

Trypsin inhibitory activity of the different samples was determined using Nα-benzoyl-DL-arginine-p-nitroanilide (BApNA) as a substrate. To perform the inhibitory activity experiments, we adapted the method of Erlanger, Kokowsky and Cohen [14] to 96- well plate measurements. To perform the experiments, trypsin (0.25 mg/mL) was pre-incubated with increasing concentrations of the extract (concentrations ranging from 0 to 50 μg/mL) in 100 mM Tris-HCl buffer (pH 7.5) with 50 mM CaCl_2_. The samples were pre-incubated for 10 min at 37 °C, and then the BApNA substrate was added to each well at a final concentration of 1 mM. The hydrolysis of BApNA was monitored by recording increases in the absorbance at 410 nm using a Tecan Infinite M200 PRO spectrophotometer (Männedorf, Switzerland). The plates were incubated at 37 °C for 10 min. One trypsin inhibitory unit (1 TIU) was defined as the decrease of 0.01 unit of absorbance at 410 nm per 10 min assay, at 37 °C. All experiments were carried out in triplicate.

### 2.5. Heat Treatment and Affinity Chromatography Purification

According to our previous studies, it is common that protease inhibitors present high physicochemical stability with minimal loss of inhibitory activity [5]. Accordingly, in the first purification step, the crude extract (named here as YBPCE) was subjected to 100 °C for 5 min. After cooling at room temperature, thermally denatured proteins were removed by centrifugation for 30 min at 7000× *g* and 4 °C. Afterwards, the total protein content and the inhibitory activity of the non-treated crude extract and heat-treated sample (named hereafter as YBPHT) were evaluated.

For protein purification, a sample of YBPHT containing 40.4 ± 0.1 μg/mL of protein was loaded to a Trypsin-glyoxyl-agarose column (1.5 × 10 cm) previously equilibrated with 0.1 M Tris-HCl buffer (pH 8.0) containing 0.2 M NaCl. This protocol was optimized previously by our group [15]. In the first step of purification, the unbound proteins were eluted with equilibration buffer, and then affiliated proteins were eluted with HCl pH 2.0. The eluted fractions were adjusted to pH 7.0 with 0.1 M NaOH, and the fractions exhibiting trypsin inhibitory activity were pooled (see previous Section 2.3 Trypsin inhibition measurements).

### 2.6. Biochemical Characterization of the Inhibitor

#### 2.6.1. Inhibitory Assays and *K*i Determination

The inhibition constant (*K*i) of YBPTI was determined by performing a Dixon plot analysis (1/v vs. [I], where [I] is the inhibitor concentration). Previously, the inhibition of trypsin activity in the presence of different concentrations of the inhibitor and two different substrate concentrations was evaluated. The inhibition experiments were performed with increasing concentrations of the inhibitor YBPTI (0–3.2 μg/mL) and BApNA (1.0 and 2.0 mM). Bovine trypsin was assayed at a fixed concentration of 0.25 mg/mL, similarly to that described in [16]. The *K*i was determined from a Dixon plot, where the reciprocal of the enzyme reaction rate was expressed as 1/v. The *K*i value was derived from the intersection of the two lines plotted for two different BApNA concentrations. All the reactions were performed in triplicate.

#### 2.6.2. YBPTI Stability Studies

The physico-chemical stability of the purified YBPTI was evaluated. First, the effect of the protease inhibitor under extreme temperatures was evaluated as previously described [12]. In brief, YBPTI aliquots were incubated at 100 °C for different periods of time (i.e., 30, 60, 90, 120, 150 and 180 min). After incubation, the samples were cooled at room temperature and the residual trypsin inhibitory activity was determined as described in Section 2.4. Second, the effect of pH on YBPTI stability was studied. To perform the experiments, the residual activity after incubation at extreme pHs (pH 2 and pH 12) for 30 and 60 min at 25 °C was determined. The residual trypsin inhibitory activity of YBPTI was measured as detailed above.

### 2.7. Biological Assays

#### 2.7.1. Antimicrobial Activity against Pathogenic Bacterial and Fungal Strains

The antifungal activity of the YBPCE, YBPHT, and purified YBPTI against a set of and pathogenic microbial strains (see Section 2.2) was evaluated using the agar diffusion assay. This method was based on the Kirby–Bauer test with slight modifications [17]. In essence, for the preparation of cell cultures of *C. albicans*, an inoculum from a fungal stock was transferred to a Petri dish containing Sabouraud agar and allowed to grow at 36 °C for 2 days. The fungal suspension was adjusted with physiological solution to 0.3 McFarland scale (10^5^ cells/mL). To perform the assays, 10 µL of each test sample was placed over the previously inoculated Sabouraud agar plates. Once the drop placed in the plate was dried, the plates were then incubated at 36 °C for 24 h to allow fungal growth. After incubation, the diameters of the fungal growth inhibition were measured.

#### 2.7.2. Antimicrobial Activity against Pathogenic Candida Albicans

To further investigate the antifungal activity of YBPCE, YBPHT, and purified YBPTI on *Candida albicans* growth, the minimal inhibitory concentration (MIC) and minimal fungicidal concentration (MFC) for all these samples was determined. To perform the experiments, *C. albicans* was seeded at a concentration of 10^5^ cells/mL onto 96-well plates. The plates were then incubated at 36 °C in 200 µL microplates in the absence and presence of different sample concentrations (i.e., YBPCE: 1653.1–2.3 µg/mL; YBPHT: 687.6–0.9 µg/mL; YBPTI: 51.1–0.07 µg/mL). Fluconazole (3.5 µg/mL) was also evaluated as reference drug with known antifungal properties. The absorbance of all the wells was measured (at 620 nm) at different time points of incubation (0, 2, 4, 16, 18 and 20 h) using a Tecan Infinite M200 PRO spectrophotometer (Männedorf, Switzerland) plate reader. All the growth inhibition experiments were performed in triplicate. The MIC was determined for each sample as the minimum sample concentration required to reduce yeast growth on 50%. The MFC was determined as the minimum sample concentration required to produce complete growth inhibition. After 20 h of incubation in the presence of these compounds, yeast cells were visualized using an optical microscope at 1000× magnification (Nikon Eclipse OPT-01514) by direct observation and with 30 min incubation with methylene blue (1:1 relation). The yeast cells grown in the absence of YBPTI were also determined as the control condition.

#### 2.7.3. Plasma Membrane Permeabilization Experiments

Plasma membrane permeabilization experiments were performed by investigating SYTOX Green (Molecular Probes Invitrogen, EUA) uptake, as described previously by [18] with some modifications. Briefly, a culture of *C. albicans* (10^5^ cells/mL) was incubated in the absence or in the presence of YBPTI at the concentration of 5.7 µg/mL for 20 h. Aliquots of the suspension of yeast cells were incubated with 0.2 µM SYTOX Green (1:1 ratio) for 20 min at 25 °C. Afterwards, the cells were observed in an optical microscope (IMLD Biosystems) equipped with a fluorescence filter set for fluorescein detection (excitation wavelengths: 450–490 nm; emission wavelength: 500 nm). Both negative and positive in the absence of peptide or with 3.5 µg/mL fluconazole, respectively, were performed as control conditions.

#### 2.7.4. Hypoglycemic Activity

The α-Glucosidase inhibitory activity was evaluated by the [19] method, with slight modifications. The original protocol was adapted to a 96- well plate measurements, using the substrate 4-nitrophenol-α-D-glucopyranoside (PNPG). In brief, a fixed amount of α-glucosidase (0.5 U/mL) was preincubated with different concentrations of the inhibitor (ranging from 0 to 689 ng/mL) in 100 mM sodium phosphate buffer (pH 7.4). After 10 min pre-incubation at 37 °C, the substrate was added to each reaction mixture at a final concentration of 0.2 mM. The hydrolysis of PNPG was recorded through the increase in the absorbance at 405 nm at 37 °C every minute for 20 min. All the measurements were carried out in triplicate.

#### 2.7.5. Cytotoxicity Assays

The cytotoxicity of the purified trypsin inhibitor toward Hela (ATCC CCL-2) cells was evaluated using a MTT assay, similarly as described elsewhere [20,21]. To perform the experiments, the cells were seeded in 96-well plates at a concentration of 1.0 × 10^4^ cells per well and incubated for 24 h. Afterwards, the cells were treated with the indicated inhibitor concentrations. After 24 h of incubation, aliquots of an MTT solution (0.5 mg/mL) were added to each well. The plates were then incubated for an additional 3 h at 37 °C. After incubation, the supernatant was removed and 100 µL of DMSO were added to each well. The absorbance at 540 nm of each well was measured using a UV-vis microplate reader MultiSkan FC (Thermo fisher Scientific, Waltham, MA, USA).

## 3. Results and Discussion

### 3.1. Isolation and Purification of YBPTI

The yellow bell pepper (*Capsicum annuum* L.) belongs to the genus *Capsicum* (vegetable pepper). Different species from this genus are commonly used as food products, either as fresh vegetables or as processed foods [22]. During the last few years, different reports have suggested that *Capsicum annuum* seeds are a rich source of protease inhibitors [11,23,24,25,26]. These reports demonstrated the presence of mainly serine proteinase inhibitors such as trypsin and chymotrypsin. Examples of such inhibitors are: PSI-1.1 and PSI-1.2, which have been isolated from paprika seeds [23], CaTI isolated from chilli pepper seeds [24,25], and CapA1 and CapA2 obtained from the leaves of *Capsicum annum* var. Phule Jyoti. More recently, PIJP was isolated from jalapeño pepper [26].

Since yellow bell pepper is a rich source of protease inhibitors, we prepared a crude extract of *Capsicum annuum* L. seeds (hereafter named as YBPCE) to evaluate its trypsin inhibitory capacity. The protein concentration of the sample was 802.4 ± 1.12 mg/mL (see Table 1), which is in agreement with previous studies [11]. We have next evaluated the trypsin inhibitory activity of the crude extract. The presence of the extract in the reaction caused a rapid decrease in the trypsin activity, showing the specific inhibitory activity of 0.55 TIU/mg. The effect of temperature (100 °C) on the trypsin inhibitory activity of YBPCE is shown Table 1. After treatment, we observed a significant increase in the specific inhibitory activity of the sample between the heated to 100 °C and unheated extract, suggesting that such a trypsin inhibitor is a thermostable molecule. In agreement, we observed a 2.1-fold of purification after heat treatment, suggesting that other non-thermostable proteins have been removed.

**Figure 1:**
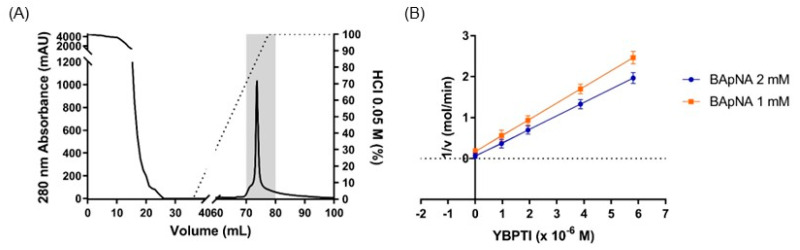
Purification and kinetic characterization of Yellow Bell Pepper trypsin inhibitor (YBPTI). (**A**) Affinity chromatography on immobilized trypsin. Fractions with trypsin inhibitory activity (grey area) were pooled and named YBPTI, (**B**) Dixon plot (1/v vs. [I]) for identification of the Ki. Each point represents the mean of three estimates.

After the initial characterization, we decided to perform further purification of the trypsin inhibitor found in the yellow pepper seeds. For this purpose, we selected the heat-treated fraction, and we performed a high-speed centrifugation step to clarify the sample (see Materials and Methods for details). The resultant sample was applied to single-step purification based on a glyoxyl-agarose matrix prepared in house, containing the target enzyme covalently immobilized on the resin. After purification by affinity chromatography, the trypsin inhibitory activity of the eluted fractions was evaluated. As shown in Figure 1A, the purified inhibitor eluted in a single sharp peak with a specific inhibitory activity against trypsin of 77.29 TIU/mg (Table 1). The high specificity and efficiency of the purification method allowed us to obtain a highly purified sample of the trypsin inhibitor from yellow pepper seeds suitable for further inhibition kinetics characterization.

### 3.2. Inhibition Kinetics and Physicochemical Properties of YBPTI

Inhibition kinetic studies of YBPTI against trypsin activity was carried out following a protocol developed in our laboratory [12]. Analysis of the data revealed that YBPTI has an IC_50_ value of 3.9 µg/mL (2.05 × 10^−7^ M) and a *K*i value of 1.7 × 10^−6^ M (see Figure 1B). The *K*i value obtained for this inhibitor is in the range of other previously described trypsin inhibitors purified from natural sources, such as the protease inhibitor isolated from *B. microplus* larvae (*Ki* = 1.20 × 10^−7^ M) [27], the Kunitz inhibitor isolated from *Boophilus microplus* (*Ki* = 1.08 × 10^−7^ M) [28], or the TcTI trypsin inhibitor obtained from *Torresea cearensis* (*Ki* = 1.4 × 10^−6^ M) [29], among others.

**Figure 2:**
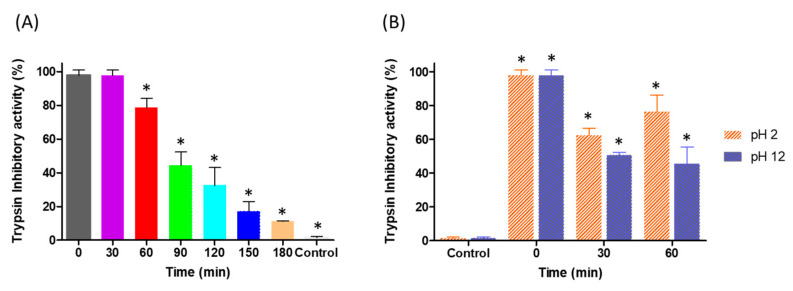
Physicochemical stability of YBPTI. (**A**) Effect of temperature and (**B**) pH treatment on the trypsin inhibitory activity of YBPTI. In (**A**), the asterisk (*) indicates significantly different compared to time 0. In (**B**), the asteric (*) indicates significantly different compared to control conditions. In both cases, a one-way ANOVA (*p <* 0.05) and Tukeýs multiple comparison test was performed.

To evaluate the stability of YBPTI at different temperatures and pHs, the protein was incubated at 100 °C for various amounts of time and at pH 2 and 12 for 30 and 60 min at 25 °C. The residual trypsin inhibitory activity was then assessed. As shown in Figure 2A, after incubating for 30 min at 100 °C, the residual trypsin inhibitory activity of the YBPTI was 99.67 ± 5.55%; that is, almost all of its activity was maintained. Interestingly, after one hour of exposure to this extreme temperature, the YBPTI sample maintained 74.65 ± 4.03% of its trypsin inhibitory activity, which makes this peptide inhibitor a remarkable, highly stable molecule. Based on these findings, we can conclude that YBPTI was found to be stable at extreme pHs, retaining approximately 60% of its activity after 1 h of incubation at both pH 2 and 12.

There are very few studies that have investigated the thermal stability of proteins at 100 °C. Previously, a reduced number of PIs with high stability at extreme temperatures and pHs have been studied. Most of these inhibitors showed interesting biological activities, such as potential as biopesticides, with inhibitory activity on insect intestinal proteases or inhibition of larval growth: HSTI [30], C11PI [31], CFPI [32] and RsBBI1 [33]. Other previously reported biological activities for these inhibitors include antibacterial activity against *S. aureus* by LzaBBI [34] and anticoagulant activity in the extrinsic coagulation pathway by MpBBI [35]. Despite the great pharmaceutical potential of these highly stable plant-derived inhibitors, the biological activities of these PIs have been little explored.

Overall, our results show that YBPTI is a highly stable trypsin inhibitor, with few reports of other proteins with similar stability at extreme temperatures and pHs. This stability, combined with its trypsin inhibitory activity, makes YBPTI a potentially useful protein for pharmaceutical applications.

### 3.3. Antifungal Activity of YBPTI

**Figure 3:**
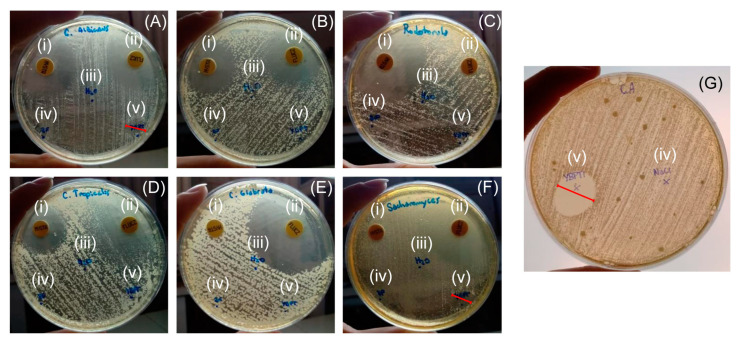
Growth inhibition of fungal strains by YBPTI. The growth inhibition of seven fungal strains was evaluated by agar diffusion: (**A**) *C. albicans*, (**B**) *C. krusei*, (**C**) *Rodothorula*, (**D**) *C. tropicalis*, (**E**) *C. glabrata*, (**F**) *S. cerevisiae*. In (**A**–**F**) 5 µg of *YBPTI* were tested. In (**G**), *C. albicans* was assayed with 66 µg YBPTI. The treatments are: (i) Nystatin, (ii) Fluconazole, (iii) distilled water, (iv) 100 mM phosphate buffer pH 7.4, (v) YBPTI.

The antifungal activity of YBPTI was evaluated against a set of fungal strains. As shown in Figure 3, a clear area of growth inhibition was observed for *C. albicans* and *S. cerevisiae*, tested with 5 µg of YBPTI, and an inhibition halo of 21 mm was observed for *C. albicans*, when 66 µg of YBPTI were added to the plate. Interestingly, no inhibitory effect of the *Capsicum annuum* L. inhibitor was observed for other fungal or bacterial strains (Figure 3 and Table S1).

Given that *C. albicans* is a pathogen of great importance in clinics, we decided to further investigate the inhibitory activity of YBPTI against this microorganism. The effect of the purified YBPTI was evaluated using serial dilutions in SB broth by adding increasing sample concentrations. The minimum inhibitory concentration (MIC) was determined to be 5.7 µg/mL, showing a fungicidal effect of MFC/MIC < 4 against this pathogen. Under similar experimental conditions, fluconazole showed a MIC of 3.5 μg/mL, which is certainly close to the MIC observed for YBPTI obtained from yellow pepper seeds.

**Figure 4:**
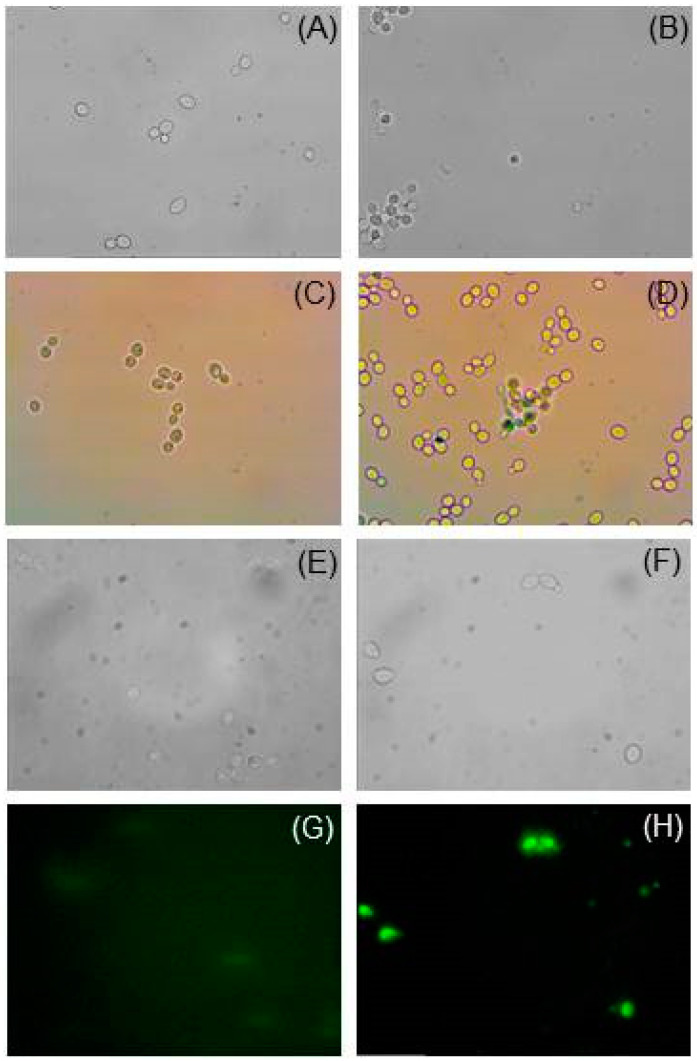
Effect of YBPTI on *C. albicans* cell morphology. (**A**) Representative optical microscopy images of control *C. albicans,* and (**B**) the same cells treated with YBPTI (5.7 µg/mL). (**C**) *C. albicans* stained with methylene blue (**C**) in the absence or (**D**) presence of YBPTI (5.7 µg/mL). (**E**–**H**) *C. albicans* permeabilization assay using SYTOX green. Representative optical microscopy images of control *C. albicans* stained with SYTOX green in (**E**) presence or (**F**) absence of YBPTI (5.7 µg/mL). (**G**,**H**) shows fluorescence microscopy images of (**E**,**F**), respectively. In (**E**,**F**), SYTOX fluorescence emission is shown in green.

Next, we performed a morphological analysis by light microscopy to confirm the toxic effect of the YBPTI on *C. albicans* (Figure 4). Through direct observation it was possible to demonstrate the presence of agglomerated and darkened cells (Figure 4B), unlike the control cells that showed normal fungal growth and morphology (Figure 4A). Then, SYTOX Green was used to confirm that YBPTI is able to disrupt the plasma membrane of yeast cells and cause cell death. Overall, these results suggest that YBPTI from *Capsicum annuum* L. has the potential to be developed as a natural drug for treating infections caused by *C. albicans.*

As mentioned above, many pathogenic fungi produce extracellular proteases that can play an active role in the development of diseases. Virulence and optimal growth of fungi depend on several secreted extracellular proteases, among which serine proteases are of particular interest. This proteolytic system allows fungi to survive and penetrate tissues. One of the first examples of this phenomenon was studied for the first time in tomatoes infected with *Phytophthora infestans* [36], in which the increase in trypsin levels and trypsin inhibitors were correlated with plant resistance to the pathogen. In recent years, an increasing number of antimicrobial peptides rich in cysteine residues have been isolated from plants, particularly from seeds, such as *Abelmoschus moschatus* [37]. Indeed, previous studies on other varieties of *Capsicum annuum* L. have also shown that they have peptide inhibitors with antifungal activity due to their plasma membrane permeabilizing capacity [24,25]. Similarly, other serine and metalloprotease inhibitors have been studied and have been reported to possess antimicrobial activities [10].

### 3.4. Hypoglycemic Activity of YBPTI

Among the risk groups that are more susceptible to infection by *Candida* spp. are patients with diabetes mellitus (DM) [38], whose infections are aggravated in cases of uncontrolled hyperglycemia [39]. For this reason, it has been previously suggested that regulating blood glucose levels may be an important factor for the prevention and treatment of comorbid invasive candida infections.

**Figure 5:**
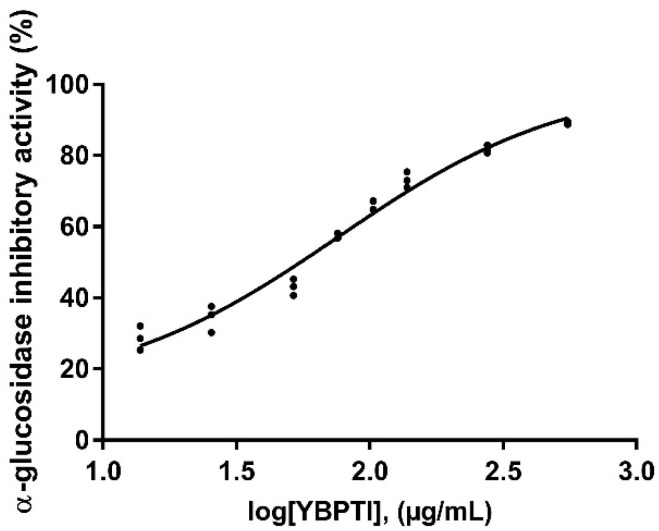
Effect of YBPTI on the α-glucosidase activity. YBPTI showed a concentration-dependent inhibition of α-glucosidase activity. Note that the derived IC50 value is 75.33 ± 1.17 ng/mL.

In this study, we have further investigated the inhibitory activity YBPTI against α-1,4-glucosidase. As shown in Figure 5, YBPTI was able to inhibit the enzyme α-1,4-glucosidase with an IC50 value of 75.33 ± 1.17 ng/mL. It is expected that inhibiting the activity of α-1,4-glucosidase leads to a decrease in the release of free glucose from complex carbohydrates, thereby lowering local blood glucose levels [40]. Indeed, hypoglycemic-lowering activity tests have been rarely described for PIs, being mostly reported for peptides derived from protein hydrolysates [41,42]. In this regard, peptides from whey protein hydrolysates have been shown to have strong α-1,4-glucosidase inhibitory activity, with an IC50 of 3.5 mg/mL, according to research by Konrad et al. [41]. Similar levels of inhibition of α-1,4-glucosidase were reported by Matsui et al. [43] and Yu et al. [44] for different peptides.

### 3.5. Cytotoxicity of YBPTI against HeLa Cells

**Figure 6:**
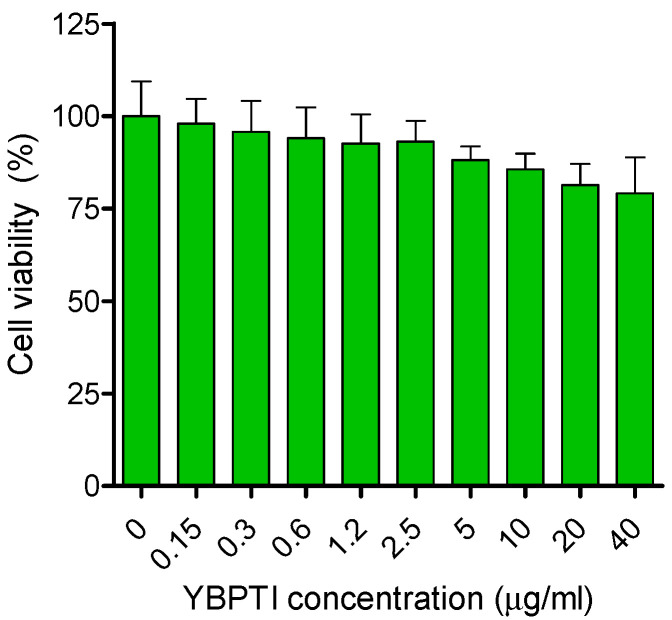
Cytotoxicity of YBPTI against HeLa cells. Effect of YBPTY on the viability of HeLa cells after 24 h incubation.

To evaluate the toxicity of YBPTI against human cells, in vitro cytotoxicity assays with HeLa cells was carried out, similarly to those described previously for other bioactive molecules [45,46]. These assays were performed using the range of concentrations where YBPTI inhibited the growth of *Candida albicans* in the range 0.15 µg/mL to 40 µg/mL. As shown in Figure 6, a value of around 5 µg/mL would maintain almost 90% cell viability while a value of 40 µg/mL would maintain close to 80% cell viability. Thus, the approximated IC50 is certainly higher than 50 µg/mL, a value almost 10-fold over the observed MIC determined against *C. albicans*.

On the basis of these findings, we can state that our inhibitor did not show a reduction in cell viability at the assayed inhibitor concentrations in the range 0.15 to 40 μg/mL, which demonstrates the safety of this natural compound for the potential treatment of *C. albicans* infections, as well as other pharmaceutical applications.

## 4. Conclusions

The increasing antifungal resistance among pathogenic fungi is a serious global concern. The limited number of currently available drugs to treat invasive fungal infections has led to the emergence of increasingly resistant strains, leaving few therapeutic options. Targeting virulence factors, such as extracellular proteases, is a promising strategy for the development of novel antifungal drugs.

Herein, we report the purification and characterization of a thermostable trypsin inhibitor from *Capsicum annuum* L. seeds with dual antifungal and hypoglycemic properties. This natural inhibitor was obtained from Yellow Bell Pepper (*Capsicum annuum* L.) seeds by heat-treatment and affinity purification with immobilized trypsin. The purified inhibitor showed potent and specific activity in vitro against pathogenic *Candida albicans*, and was found to be safe and non-toxic against human cells. Additionally, the inhibitor also exhibited α-1,4-glucosidase inhibition activity, positioning this inhibitor as one of the first plant-derived molecules with such a particular dual combination of biological activities. Our results demonstrate that this novel inhibitor could potentially be used as an antifungal agent in pharmaceutical preparations to prevent invasive candida infections.

## Figures and Tables

**Table 1:** Trypsin inhibitory activity.

Purification Step	Total Protein Amount (mg)	Total Inhibitory Activity (TIU) ^a^	Specific Inhibitory Activity (TIU/mg)	Purity (Fold) ^b^	Yield (%) ^c^
Crude extract	80.24 ± 0.17	44 ± 0.05	0.55 ± 0.01	1	100
100 eat treatment	35.53 ± 0.06	39.4 ± 0.05	1.11 ± 0.01	2.1	89.5
Affinity chromatography	0.48 ± 0.02	37.1 ± 0.03	77.29 ± 0.06	140.5	84.3

^a^ One trypsin inhibitory unit (1 TIU) was defined as the amount of inhibitor that decreased absorbance at 410 nm by 0.1 under the assay conditions; ^b^ The purification index (Purity) was calculated as the ratio between the specific inhibitory activity determined after each purification step as compared to the initial inhibitory activity present in the crude extract. ^c^ Yield of inhibitory activity after each purification step compared to the crude extract (%).

## Data Availability

The data presented in this study are available in this article or in the associated Supporting Information file.

## Supplementary Materials

The following are available online at https://www.mdpi.com/article/10.3390/pharmaceutics15030781/s1, Table S1: Evaluation of the antibacterial activity of *Capsicum annuum* L. crude extract (YBPCE) and heat-treated sample (YBPHT).

## References

[1] Kainz K., Bauer M.A., Madeo F., Carmona-Gutierrez D. (2020). Fungal Infections in Humans: The Silent Crisis. Microb. Cell.

[2] Lee Y., Puumala E., Robbins N., Cowen L.E. (2021). Antifungal Drug Resistance: Molecular Mechanisms in Candida Albicans and Beyond. Chem. Rev..

[3] Burchacka E., Pięta P., Łupicka-Słowik A. (2022). Recent Advances in Fungal Serine Protease Inhibitors. Biomed. Pharmacother..

[4] Otvos R.A., Still K.B.M., Somsen G.W., Smit A.B., Kool J. (2019). Drug Discovery on Natural Products: From Ion Channels to NAChRs, from Nature to Libraries, from Analytics to Assays. SLAS DISCOVERY Adv. Life Sci. RD.

[5] Cotabarren J., Lufrano D., Parisi M.G., Obregón W.D. (2020). Biotechnological, Biomedical, and Agronomical Applications of Plant Protease Inhibitors with High Stability: A Systematic Review. Plant Sci..

[6] Cisneros J.S., Cotabarren J., Parisi M.G., Vasconcelos M.W., Obregón W.D. (2020). Purification and Characterization of a Novel Trypsin Inhibitor from Solanum Tuberosum Subsp. Andigenum Var. Overa: Study of the Expression Levels and Preliminary Evaluation of Its Antimicrobial Activity. Int. J. Biol. Macromol..

[7] Cotabarren J., Claver S., Payrol J.A., Garcia-pardo J., Obregón W.D. (2021). Purification and Characterization of a Novel Thermostable Pa- Pain Inhibitor from Moringa Oleifera with Antimicrobial and anticoagulant Properties. Pharmaceutics.

[8] Gutierrez-Gongora D., Geddes-Mcalister J. (2021). From Naturally-Sourced Protease Inhibitors to New Treatments for Fungal Infections. J. Fungi.

[9] Shamsi T.N., Parveen R., Fatima S. (2016). Characterization, Biomedical and Agricultural Applications of Protease Inhibitors: A Review. Int. J. Biol. Macromol..

[10] Kim J.Y., Park S.C., Hwang I., Cheong H., Nah J.W., Hahm K.S., Park Y. (2009). Protease Inhibitors from Plants with Antimicrobial Activity. Int. J. Mol. Sci..

[11] Cotabarren J., Tellechea M.E., Avilés F.X., Lorenzo Rivera J., Obregón W.D. (2018). Biochemical Characterization of the YBPCI Miniprotein, the First Carboxypeptidase Inhibitor Isolated from Yellow Bell Pepper (*Capsicum Annuum* L). A Novel Contribution to the Knowledge of Miniproteins Stability. Protein Expr. Purif..

[12] Cotabarren J., Broitman D.J., Quiroga E., Obregón W.D. (2020). GdTI, the First Thermostable Trypsin Inhibitor from Geoffroea Decorticans Seeds. A Novel Natural Drug with Potential Application in Biomedicine. Int. J. Biol. Macromol..

[13] Bradford M.M. (1976). A Rapid and Sensitive Method for the Quantitation of Microgram Quantities of Protein Utilizing the Principle of Protein-Dye Binding. Anal. Biochem..

[14] Erlanger B.F., Kokowsky N., Cohen W. (1961). The Preparation and Properties of Two New Chromogenic Substrates of Trypsin. Arch. Biochem. Biophys..

[15] Obregón W.D., Ghiano N., Tellechea M., Cisneros J.S., Lazza C.M., López L.M.I., Avilés F.X. (2012). Detection and Characterisation of a New Metallocarboxypeptidase Inhibitor from Solanum Tuberosum Cv. Desirèe Using Proteomic Techniques. Food Chem..

[16] Tellechea M., Garcia-Pardo J., Cotabarren J., Lufrano D., Bakas L., Avil?s F., Obregon W., Lorenzo J., Tanco S. (2016). Microplate Assay to Study Carboxypeptidase A Inhibition in Andean Potatoes. Bio-Protocol.

[17] Bauer A.W., Kirby W.M., Sherris J.C., Turck M. (1966). Antibiotic Susceptibility Testing by a Standardized Single Disk Method. Am. J. Clin. Pathol..

[18] Thevissen K., Terras F.R.G., Broekaert W.F. (1999). Permeabilization of Fungal Membranes by Plant Defensins Inhibits Fungal Growth. Appl. Environ. Microbiol..

[19] Kim Y.M., Wang M.H., Rhee H.I. (2004). A Novel α-Glucosidase Inhibitor from Pine Bark. Carbohydr. Res..

[20] Mosmann T. (1983). Rapid Colorimetric Assay for Cellular Growth and Survival: Application to Proliferation and Cytotoxicity Assays. J. Immunol. Methods.

[21] Twentyman P.R., Luscombe M. (1987). A Study of Some Variables in a Tetrazolium Dye (MTT) Based Assay for Cell Growth and Chemosensitivity. Br. J. Cancer.

[22] Anaya-Esparza L.M., de la Mora Z.V., Vázquez-Paulino O., Ascencio F., Villarruel-López A. (2021). Bell Peppers (*Capsicum Annum* L.) Losses and Wastes: Source for Food and Pharmaceutical Applications. Molecules.

[23] Antcheva N., Pintar A., Patthy A., Simoncsits A., Barta E., Tchorbanov B., Pongor S. (2001). Proteins of Circularly Permuted Sequence Present within the Same Organism: The Major Serine Proteinase Inhibitor from Capsicum Annuum Seeds. Protein Sci..

[24] Ribeiro S.F.F., Silva M.S., Da Cunha M., Carvalho A.O., Dias G.B., Rabelo G., Mello É.O., Santa-Catarina C., Rodrigues R., Gomes V.M. (2012). *Capsicum Annuum* L. trypsin Inhibitor as a Template Scaffold for New Drug Development against Pathogenic Yeast. Antonie van Leeuwenhoek. Int. J. Gen. Mol. Microbiol..

[25] Ribeiro S.F.F., Fernandes K.V.S., Santos I.S., Taveira G.B., Carvalho A.O., Lopes J.L.S., Beltramini L.M., Rodrigues R., Vasconcelos I.M., Da Cunha M. (2013). New Small Proteinase Inhibitors from Capsicum Annuum Seeds: Characterization, Stability, Spectroscopic Analysis and a CDNA Cloning. Biopolymers.

[26] Tamhane V.A., Chougule N.P., Giri A.P., Dixit A.R., Sainani M.N., Gupta V.S. (2005). In Vivo and in Vitro Effect of *Capsicum Annum* Proteinase Inhibitors on *Helicoverpa Armigera* Gut Proteinases. Biochim. Biophys. Acta–Gen. Subj..

[27] Tanaka A.S., Andreotti R., Gomes A., Torquato R.J.S., Sampaio M.U., Sampaio C.A.M. (1999). A Double Headed Serine Proteinase Inhibitor—Human Plasma Kallikrein and Elastase Inhibitor—from *Boophilus Microplus* Larvae. Immunopharmacology.

[28] Sasaki S.D., de Lima C.A., Lovato D.V., Juliano M.A., Torquato R.J.S., Tanaka A.S. (2008). BmSI-7, a Novel Subtilisin Inhibitor from *Boophilus Microplus*, with Activity toward Pr1 Proteases from the Fungus *Metarhizium Anisopliae*. Exp. Parasitol..

[29] Sampaio C.A., Oliva M.L., Tanaka A.S., Sampaio M.U. (1991). Proteinase Inhibitors in Brazilian Leguminosae. Memórias Do Inst. Oswaldo Cruz.

[30] Aguirre C., Valdés-Rodríguez S., Mendoza-Hernández G., Rojo-Domínguez A., Blanco-Labra A. (2004). A Novel 8.7 KDa Protease Inhibitor from Chan Seeds (*Hyptis Suaveolens* L.) Inhibits Proteases from the Larger Grain Borer *Prostephanus Truncatus* (Coleoptera: Bostrichidae). Comp. Biochem. Physiol. B Biochem. Mol. Biol..

[31] Swathi M., Lokya V., Swaroop V., Mallikarjuna N., Kannan M., Dutta-Gupta A., Padmasree K. (2014). Structural and Functional Characterization of Proteinase Inhibitors from Seeds of *Cajanus Cajan* (Cv. ICP 7118). Plant Physiol. Biochem..

[32] Dantzger M., Vasconcelos I.M., Scorsato V., Aparicio R., Marangoni S., Macedo M.L.R. (2015). Bowman-Birk Proteinase Inhibitor from *Clitoria Fairchildiana* Seeds: Isolation, Biochemical Properties and Insecticidal Potential. Phytochemistry.

[33] Mohanraj S.S., Tetali S.D., Mallikarjuna N., Dutta-Gupta A., Padmasree K. (2018). Biochemical Properties of a Bacterially-Expressed Bowman-Birk Inhibitor from Rhynchosia Sublobata (Schumach.) Meikle Seeds and Its Activity against Gut Proteases of Achaea Janata. Phytochemistry.

[34] Martins T.F., Vasconcelos I.M., Silva R.G.G., Silva F.D.A., Souza P.F.N., Varela A.L.N., Albuquerque L.M., Oliveira J.T.A. (2018). A Bowman-Birk Inhibitor from the Seeds of Luetzelburgia Auriculata Inhibits Staphylococcus Aureus Growth by Promoting Severe Cell Membrane Damage. J. Nat. Prod..

[35] Lazza C., Trejo S., Obregon W., Pistaccio L., Caffini N., Lopez L. (2010). A Novel Trypsin and α-Chymotrypsin Inhibitor from Maclura Pomifera Seeds. Lett. Drug Des. Discov..

[36] Woloshuk C.P., Meulenhoff J.S., Sela-Buurlage M., Van Den Elzen P.J.M., Cornelissen B.J.C. (1991). Pathogen-Induced Proteins with Inhibitory Activity toward Phytophthora Infestans. Plant Cell.

[37] Dokka M.K., Davuluri S.P. (2014). Antimicrobial Activity of a Trypsin Inhibitor from the Seeds of *Abelmoschus Moschatus* L.. Int. J. Curr. Microbiol. Appl. Sci..

[38] Mohammed L., Jha G., Malasevskaia I., Goud H.K., Hassan A. (2021). The Interplay Between Sugar and Yeast Infections: Do Diabetics Have a Greater Predisposition to Develop Oral and Vulvovaginal Candidiasis?. Cureus.

[39] Rodrigues C.F., Rodrigues M.E., Henriques M. (2019). Candida Sp. Infections in Patients with Diabetes Mellitus. J. Clin. Med..

[40] Jaiswal N., Bhatia V., Srivastava S.P., Srivastava A.K., Tamrakar A.K. (2012). Antidiabetic Effect of Eclipta Alba Associated with the Inhibition of Alpha-Glucosidase and Aldose Reductase. Nat. Prod. Res..

[41] Konrad B., Anna D., Marek S., Marta P., Aleksandra Z., Józefa C. (2014). The Evaluation of Dipeptidyl Peptidase (DPP)-IV, α-Glucosidase and Angiotensin Converting Enzyme (ACE) Inhibitory Activities of Whey Proteins Hydrolyzed with Serine Protease Isolated from Asian Pumpkin (Cucurbita Ficifolia). Int. J. Pept. Res. Ther..

[42] Wang R., Zhao H., Pan X., Orfila C., Lu W., Ma Y. (2019). Preparation of Bioactive Peptides with Antidiabetic, Antihypertensive, and Antioxidant Activities and Identification of α-Glucosidase Inhibitory Peptides from Soy Protein. Food Sci. Nutr..

[43] Matsui T., Oki T., Osajima Y. (1999). Isolation and Identification of Peptidic α-Glucosidase Inhibitors Derived from Sardine Muscle Hydrolyzate. Z. Für Naturforsch. Sect. C J. Biosci..

[44] Yu Z., Yin Y., Zhao W., Yu Y., Liu B., Liu J., Chen F. (2011). Novel Peptides Derived from Egg White Protein Inhibiting Alpha-Glucosidase. Food Chem..

[45] Covaleda-Cortés G., Hernández M., Trejo S.A., Mansur M., Rodríguez-Calado S., García-Pardo J., Lorenzo J., Vendrell J., Chávez M.A., Alonso-del-Rivero M. (2019). Characterization, recombinant production and structure-function analysis of NvCI, A picomolar metallocarboxypeptidase inhibitor from the marine snail nerita versicolor. Mar. Drugs.

[46] Maculins T., Garcia-Pardo J., Skenderovic A., Gebel J., Putyrski M., Vorobyov A., Busse P., Varga G., Kuzikov M., Zaliani A. (2020). Discovery of protein-protein interaction inhibitors by integrating protein engineering and chemical screening platforms. Cell Chem. Biol..

