# Supplementary material for: A Multifunctional Trypsin Protease Inhibitor from Yellow Bell Pepper Seeds: Uncovering Its Dual Antifungal and Hypoglycemic Properties
Source: Pharmaceutics. 2023 Feb 27;15(3):781. doi: 10.3390/pharmaceutics15030781 (PMC10054557; doi:10.3390/pharmaceutics15030781)
Supplement: Supplementary file 1 [file pharmaceutics-15-00781-s001.zip › pharmaceutics-2207767-supplementary.pdf]

SUPPORTING INFORMATION

A multifunctional Trypsin Protease Inhibitor from Yellow Bell Pepper Seeds: Uncovering its Dual Antifungal and Hypogly-cemic Properties

Juliana Cotabarren [1,a,*], Brenda Ozón [1,a], Santiago Claver [1], Florencia Geier [1], Martina Rossotti [1], Javier Garcia-Pardo [2,*] and Walter David Obregón [1,*]

[1] Centro de Investigación de Proteínas Vegetales (CIPROVE), Departamento de Ciencias Biológicas, Facultad de Ciencias Exactas, Universidad Nacional de la Plata, 47 y 115 s/N, B1900AVW La Plata, Buenos Aires, Argentina.

[2] Institut de Biotecnologia i Biomedicina and Departament de Bioquimica i Biologia Molecular, Universitat Autònoma de Barcelona, 08193 Bellaterra, Barcelona, Spain.

*Correspondence:

cotabarren.juliana@biol.unlp.edu.ar (J.C.); +54-221-423-5333

javiergarciapardo@msn.com (J.G-P); Tel +34-93-586-8936

davidobregon@biol.unlp.edu.ar (W.D.O.); Tel: +54-221-423-5333 (ext. 57)

SUPPLEMENTARY TABLES

Table S1: Evaluation of the antibacterial activity of *Capsicum annuum L.* crude extract (YBPCE) and heat-treated sample (YBPHT).

**Pathogenic Organism**	**Inhibition**	
**Gram negative bacteria**	**YBPCE**	**YBPHT**
Escherichia coli (ATCC 25923)	n/d	n/d
Klebsiella pneumonia (ATCC 700603)	n/d	n/d
Pseudomonas aeruginosa (ATCC 27853)	n/d	n/d
**Gram positive bacteria**		
Enterococcus faecalis (ATCC 29212)	n/d	n/d
Staphylococcus aureus (ATCC 29213)	n/d	n/d
**Fungus**		
Candida albicans (CIPROVE)	20 mm	22 mm
Candida tropicalis (CIPROVE)	17 mm	19 mm
Candida glabrata (CIPROVE)	11 mm	8 mm
Candida krusei (CIPROVE)	27 mm	28 mm
Rhodotorula spp (CIPROVE	15 mm	20 mm
Saccharomyces cerevisiae (CIPROVE)	21 mm	28 mm

[a] Abbreviations are: ATCC, American Type Culture Collection; CIPROVE, Culture collection of the CIPROVE, Facultad de Ciencias Exactas, Universidad Nacional de La Plata. NM, Not detected.
